# Case report: Diffuse large B-cell lymphoma presenting as congestive heart failure in a cat

**DOI:** 10.3389/fvets.2024.1467448

**Published:** 2024-09-25

**Authors:** Jake Johnson, Hannah Melhorn, Sonya Karchemskiy, Emily Karlin, Perry Bain, John Rush, Cornelia Peterson

**Affiliations:** Cummings School of Veterinary Medicine, Tufts University, North Grafton, MA, United States

**Keywords:** feline, cardiac neoplasia, diffuse large B-cell lymphoma, immunohistochemistry, feline immunodeficiency virus

## Abstract

Cardiac lymphoma is uncommon in cats and is rarely considered as a differential diagnosis for congestive heart failure. A 10-year-old neutered male domestic short-haired cat with clinical histories of feline immunodeficiency virus, diabetes mellitus, and congestive heart failure was humanely euthanized. Post-mortem evaluation demonstrated a massively infiltrative round cell neoplasm of the heart, resulting in CHF. Immunohistochemistry of neoplastic tissue was consistent with diffuse large B-cell lymphoma. This case demonstrates a peculiar presentation of cardiac diffuse large B-cell lymphoma, with chronic feline lentiviral infection possibly contributing to disease initiation and progression.

## Introduction

Feline congestive heart failure (CHF) is among the most common clinical presentations in small animal emergency and referral practice, with primary cardiomyopathy or left ventricular remodeling secondary to systemic disease (i.e., hyperthyroidism, systemic hypertension, chronic renal insufficiency, acromegaly) routinely identified as antecedent causes (1). Hypertrophic cardiomyopathy is the most common form of primary cardiomyopathy in the cat, with approximately 15% of the domestic population exhibiting clinical signs (2, 3). However, additional differentials for myocardial hypertrophy and pseudohypertrophy, including infiltrative processes and systemic volume depletion, should be considered in the presence of morphological abnormalities to the left ventricle during feline echocardiographic studies (4). Here, we present an unusual diagnosis in a cat presenting with findings consistent with left ventricular thickening and CHF.

## Case presentation

A 5.3 kg 10-year-old neutered male domestic short-haired cat with a two-week history of coughing was referred for suspected CHF. The medical history for this animal was significant only for poorly controlled diabetes mellitus and seropositivity to Feline Immunodeficiency Virus (FIV). On presentation, the animal was obtunded with decreased skin turgor, and the initial vital signs included: rectal temperature of 36.6°C and heart and respiratory rates of 190 beats per minute and 44 breaths per minute, respectively. Muffled heart sounds, pulmonary crackles, and increased bronchovesicular sounds in all lung fields were auscultated.

**Figure 1:**
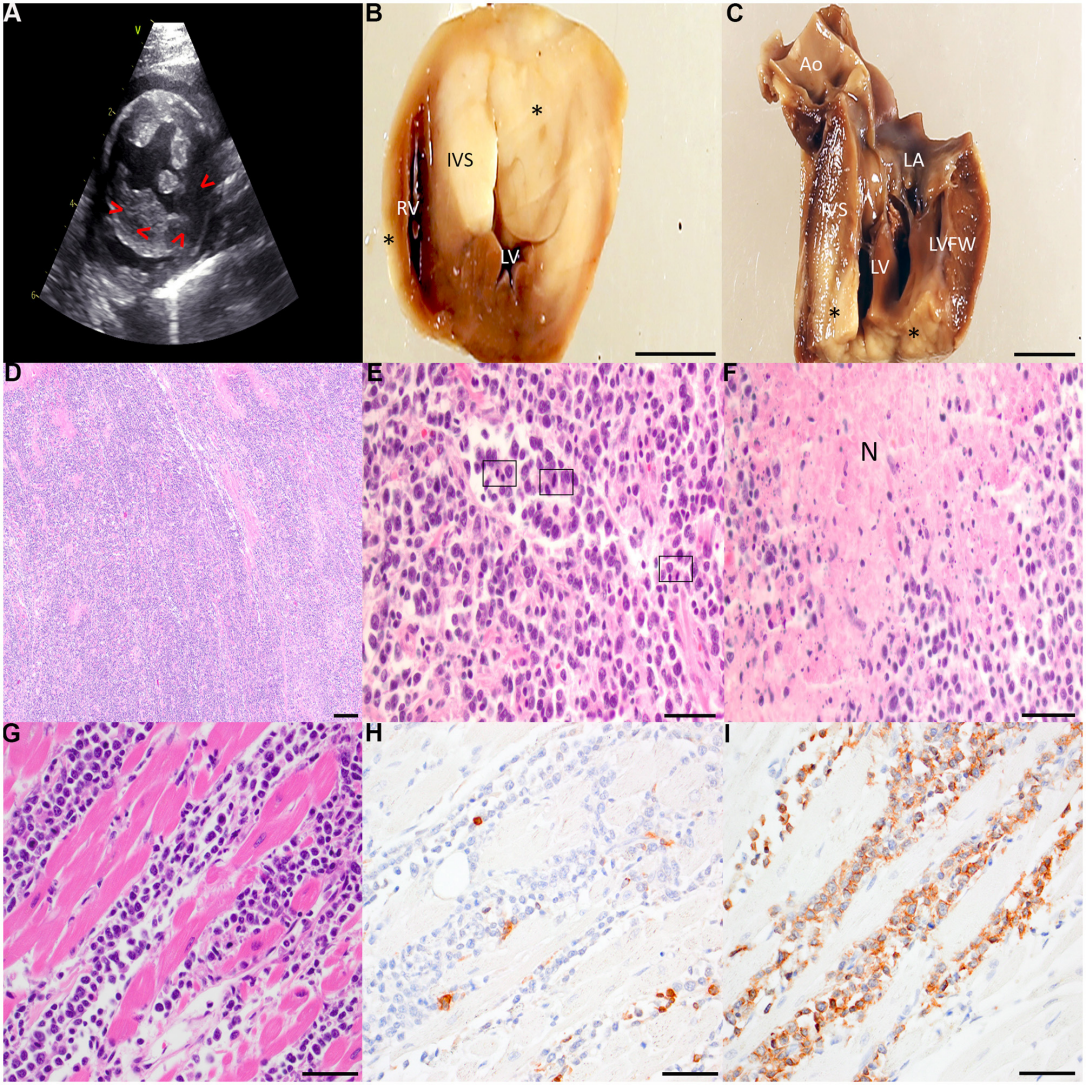
**(A)** Right parasternal short axis 2-dimensional echocardiographic image. The LVFW is mottled with mixed echogenicity. Irregularly shaped hypoechoic regions are noted in the LV myocardium (arrowheads). Left ventricular hypertrophy is present, with the IVS thickness = 0.75 cm and LVFW thickness = 0.88 cm. **(B, C)** Gross specimens of the affected heart following fixation in 10% neutral buffered formalin. The heart was sectioned transversely ~1.5 cm from the apical surface **(B)** and longitudinally through the LV inflow tract **(C)**. Several transmural, well-demarcated, non-encapsulated pale tan neoplastic nodular foci (asterisks) infiltrate the RVFW, IVS, and LVFW. Scale bar: 1 cm. Ao, aorta; IVS, interventricular septum; LA, left atrium; LV, left ventricle; LVFW, left ventricular free wall; RV, right ventricle. **(D–F)** Representative photomicrographs of hematoxylin and eosin-stained sections of affected cardiac tissue. Broad sheets of pleomorphic rounds cells efface the myocardium of the LVFW [**(D)** 40x with scale bar: 500 μm]. Large neoplastic lymphocytes infiltrate the myocardium of the RVFW with several mitotic figures (boxed regions) per high powered field [**(E)** 400x with scale bar: 50 μm]. Multifocally throughout the myocardium, but most pronounced within the IVS, are large foci of lytic necrosis (N) typified by hypereosinophilic, swollen, and fragmented myofibers admixed with abundant karyorrhectic debris and low numbers of infiltrating small lymphocytes [**(F)** 400x with scale bar: 50 μm]. **(G–I)** Representative photomicrographs of neoplastic effacement of the RVFW by large pleomorphic lymphocytes stained with hematoxylin and eosin **(G)** and immunolabled with CD3 **(H)** and CD20 **(I)**. 400x with scale bar: 50 μm.

The contemporary standard of care was provided to this animal, and informed consent was obtained from the animals' owners. Due to the persistent dependence on supplemental oxygen following diuretic therapy and thoracocentesis, a limited diagnostic echocardiogram was performed, revealing pericardial and pleural effusion, marked left atrial enlargement (left atrial to aortic ratio of 2.08), left ventricular (LV) concentric hypertrophy, and several mottled, hypoechoic lesions within the myocardium of the left ventricular free wall (LVFW) (Figure 1A).

A fluid sample was obtained during therapeutic thoracocentesis and submitted for cytopathology. The fluid was slightly hazy and straw colored prior to centrifugation (total solids: 2.0 g/dL; total nucleated cell count: 0.28 K/μL). The cytocentrifuged preparations demonstrated a mixed cell population with small lymphocytes (~40%), and a population of large lymphocytes (~30%). Neutrophils, macrophages, and plasma cells were also observed (~14%, 10%, and 6%, respectively) in addition to rare reactive mesothelial cells. Few mitotic figures were noted (Supplementary Figure I). Due to the concern for neoplastic effusion and a guarded prognosis, humane euthanasia was elected.

A necropsy was performed, and gross examination of the thorax revealed bicavitary high protein transudative effusion (pleural space: ~35 mL, total solids: 2.2g/dL; pericardial space: ~8.5 mL, total solids: 6.8 g/dL). There were multiple coalescing foci of dark red, heavy, and wet lung parenchyma which oozed serosanguinous fluid on cut section, consistent with pulmonary edema (Supplementary Figure II). Upon incising the pericardium, multiple pale tan, soft to friable nodular foci were observed infiltrating the epicardial surface. The heart weighed 31.6 g (0.59% total body weight). Upon incising the apex, the nodules exhibited transmural infiltration of the right ventricular free wall (RVFW), interventricular septum (IVS), and LVFW (Figures 1B, C).

Histopathologic examination of the heart revealed a highly cellular neoplasm comprised of sheets of moderately pleomorphic round cells infiltrating the myocardium (Figure 1D). Neoplastic cells exhibited well-defined cell borders and a high nuclear to cytoplasmic (N:C) ratio. Neoplastic cells were large, with the majority of nuclei ranging from approximately two to more than twice the size of an erythrocyte (Figure 1E). Neoplastic nuclei demonstrated one to four prominent nucleoli, and there was moderate to marked anisocytosis and anisokaryosis. There were 12 mitoses per 2.37mm^2^ (10 high powered fields). Multifocally, neoplastic cells displayed cytoplasmic swelling, hypereosinophilia, and pyknotic nuclei. The infiltrated myofibers were extensively necrotic, exhibiting mild angularity, hypereosinophilia, and fragmentation (Figure 1F). There was mild deposition of interstitial fibrillar material, and Massons trichrome staining was confirmatory for mild multifocal interstitial fibrosis (Supplementary Figure III). Routine immunohistochemistry for CD3 and CD20 was performed on sections of affected heart (Figure 1G). There were low to moderate numbers of small lymphocytes scattered throughout the neoplastic cell population which demonstrated moderate membranous immunoreactivity to CD3 (Figure 1H). The heart was effaced by sheets of large strongly CD20-positive lymphocytes (Figure 1I). Collectively, these findings supported diffuse large B-cell lymphoma (DLBCL) of the heart with resulting CHF as the ultimate cause of clinical decompensation.

## Discussion

Given the echocardiographic confirmation of LV hypertrophy and pericardial effusion in this case, an initial clinical diagnosis of decompensated LV concentric hypertrophy with CHF was made. The clinical history of poorly controlled diabetes mellitus was also considered a contributing factor due to the increased relative risk for CHF identified in diabetic cats (5, 6). However, the classic histologic features of hypertrophic cardiomyopathy (myofiber hypertrophy/disarray, pronounced interstitial fibrosis, and microvascular remodeling) were not present; the post-mortem examination, instead, demonstrated a transmurally-infiltrative neoplasm (7).

Cardiac neoplasms are rare in cats, but among them, lymphoma is the most common primary malignancy (8). Cardiac metastases, including lymphoma, are more frequently observed within the IVS and LVFW, and the clinical manifestations depend on the localization within the heart, with infiltrative processes often resulting in pericardial effusion and CHF (9–11).

Seropositivity to Feline Immunodeficiency Virus (FIV) was documented in this animal's medical history in the months preceding euthanasia. FIV is a lentivirus which results in progressive loss of CD4-positive T cells and the development of feline acquired immunodeficiency syndrome (12). FIV-infected cats are more likely to develop lymphoid tumors than non-infected cats, and lymphoma represents the majority of these neoplasms (12). B-cell lymphomas have been demonstrated in both experimental and natural FIV infections, with T-cell and non-B/non-T-cell lymphomas less commonly reported (12). Hypertrophic cardiomyopathy associated with FIV-mediated myocarditis has also been reported in a series of five animals (13).

Few case reports describing feline cardiac lymphoma exist, but to our knowledge, this is the first report detailing cardiac DLBCL with concurrent FIV seropositivity (9, 11, 14, 15). Post-mortem diagnostics in this case demonstrated DLBCL with extensive cardiac involvement; however, it remains unresolved whether this represented primary or metastatic disease, as similar neoplastic foci were observed histologically in the lung, jejunum, kidney, brain, lymph nodes (mesenteric and mesocolonic), and bone marrow (femur). While there was a history of FIV infection for this animal, further evaluation for FIV antigen within the neoplastic masses was unavailable, given the lack of tissue based FIV assays in veterinary reference and diagnostic laboratories. However, the historical seropositivity is suggestive of a virally-mediated malignant transformation of lymphocytes. The inherent constraints of echocardiographic studies performed under the suboptimal conditions on a cat with respiratory distress in an emergency room is acknowledged as an additional limitation of the current report.

In conclusion, both cardiac lymphoma and myocarditis should be considered as differential diagnoses for LV hypertrophy in cats, and in cases with echocardiographic myocardial hypertrophy and a history of FIV, appropriate molecular tests for lymphoma are recommended.

## Data Availability

The original contributions presented in the study are included in the article/Supplementary material, further inquiries can be directed to the corresponding author.
