# Supplementary material for: Case report: Diffuse large B-cell lymphoma presenting as congestive heart failure in a cat
Source: Front Vet Sci. 2024 Sep 25;11:1467448. doi: 10.3389/fvets.2024.1467448 (PMC11462620; doi:10.3389/fvets.2024.1467448)
Supplement: Supplementary file 1 [file Table_1.docx]

**Supplementary Data:**

**Supplementary Figure I.** Representative photomicrographs of the ante-mortem pleural cytology. The fluid demonstrated a high proportion (approximately 30%) of large lymphocytes. A few mitotic figures were noted, and the findings were considered to be consistent with a neoplastic effusion (A). At higher magnification, the larger lymphocytes have round nuclei with coarsely stippled chromatin, exhibit a large, prominent nucleolus, and have small to moderate amounts of deeply basophilic cytoplasm (B). Wright’s stain, scale bar: 10 µm.

**Supplementary Figure II.** Post-thoracocentesis three-view thoracic radiographs demonstrating cardiomegaly, a diffuse, patchy alveolar pulmonary pattern, and a small volume of bilateral pleural effusion (red arrows), best appreciated in the left lateral (A) and right lateral (B) views. The caudal segment of the left cranial lung lobe (red asterisks) is soft tissue opaque, and there is a cardiac shift to the left, best exhibited on the ventrodorsal (C) view. Gross thoracic evaluation exhibiting pleural (white arrow) and pericardial (white asterisk) effusion and coalescing foci of dark red and wet pulmonary parenchyma (arrowheads) on post-mortem examination (D). Histology (E) confirming the presence of homogenous, eosinophilic fluid (pulmonary edema) filling alveolar spaces. Hematoxylin and eosin stain, scale bar: 50 µm.

**Supplementary Figure III.** Left ventricular myocardium. Multifocally, thin extracellular fibrillar collagen (blue) is present within the interstitium, consistent with mild fibrosis. Massons trichrome stain, scale bar: 200 µm.
