# Supplementary figures and images for: Case report: Diffuse large B-cell lymphoma presenting as congestive heart failure in a cat
Source: Front Vet Sci. 2024 Sep 25;11:1467448. doi: 10.3389/fvets.2024.1467448 (PMC11462620; doi:10.3389/fvets.2024.1467448)

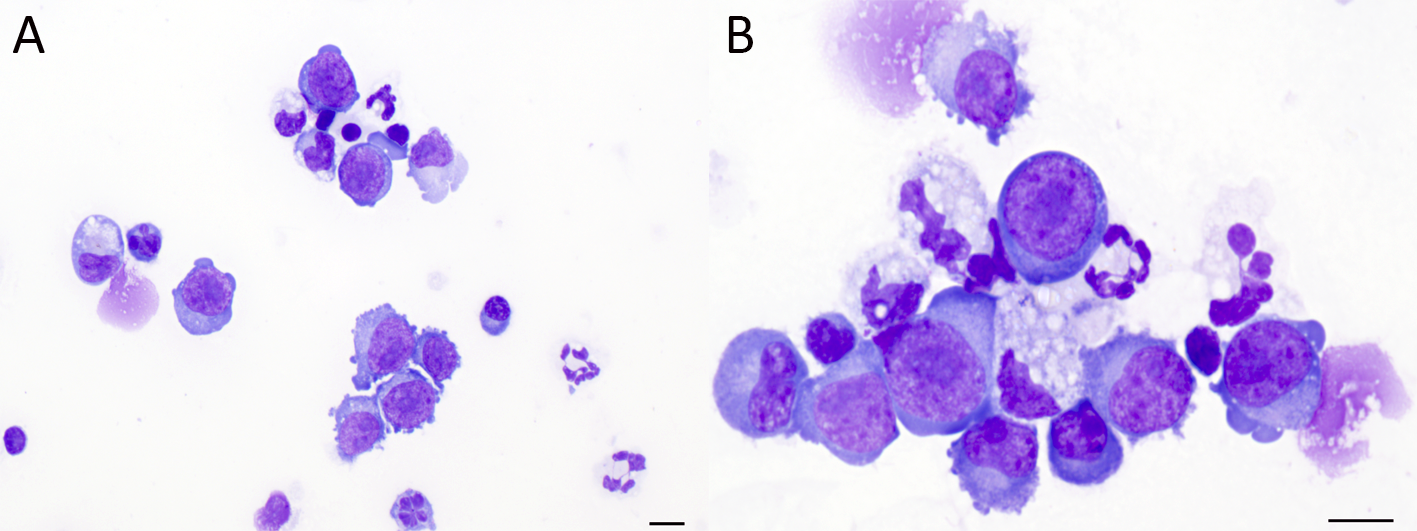

Supplement: Supplementary file 2 [file Image_1.tif]

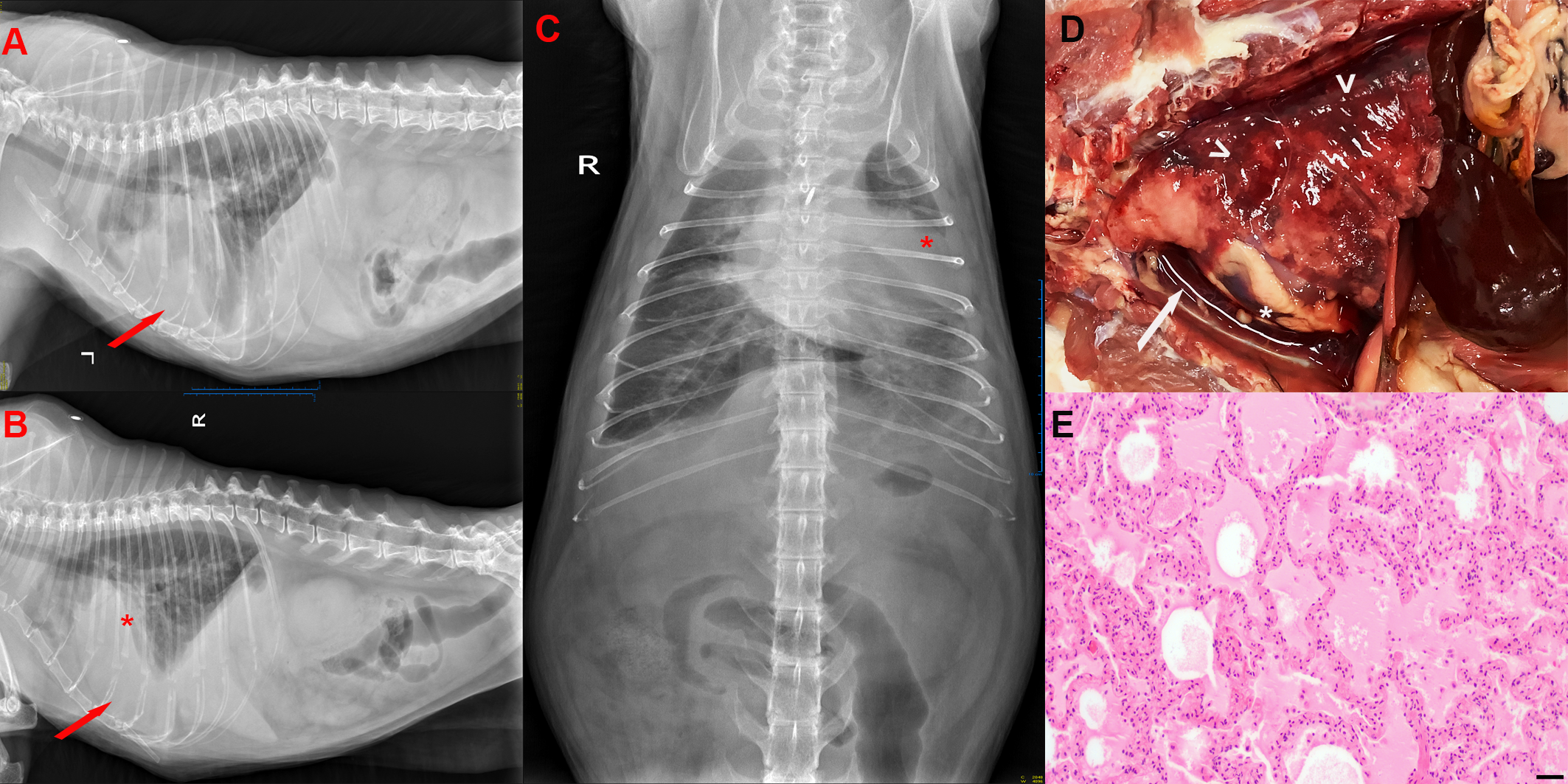

Supplement: Supplementary file 3 [file Image_2.tif]

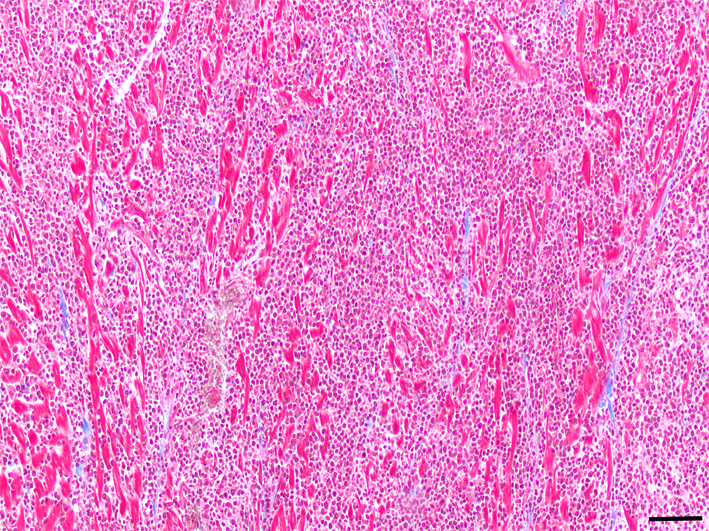

Supplement: Supplementary file 4 [file Image_3.tif]
